# Factors Associated with Tick Bite Preventive Practices among Farmworkers in Malaysia

**DOI:** 10.1371/journal.pone.0157987

**Published:** 2016-06-24

**Authors:** Masoumeh Ghane Kisomi, Li Ping Wong, Sun Tee Tay, Awang Bulgiba, Keivan Zandi, Kai Ling Kho, Fui Xian Koh, Bee Lee Ong, Tariq Jaafar, Quaza Nizamuddin Hassan Nizam

**Affiliations:** 1 Department of Social and Preventive Medicine, Faculty of Medicine, University of Malaya, Kuala Lumpur, Malaysia; 2 Julius Centre University of Malaya (JCUM), Faculty of Medicine, University of Malaya, Kuala Lumpur, Malaysia; 3 Department of Medical Microbiology, Faculty of Medicine, University of Malaya, Kuala Lumpur, Malaysia; 4 Department of Veterinary Clinical Studies, Faculty of Veterinary Medicine, University Malaysia Kelantan, Kelantan, Malaysia; 5 Department of Veterinary Services, Ministry of Agriculture and Agro-Based Industry Malaysia, Federal Government Administrative Centre, Putrajaya, Malaysia; University of Kentucky College of Medicine, UNITED STATES

## Abstract

**Background:**

Farmworkers are at high-risk for tick bites, which potentially transmit various tick-borne diseases. Previous studies show that personal prevention against tick bites is key, and certain factors namely, knowledge, experience of tick bites, and health beliefs influence compliance with tick bites preventive behaviour. This study aimed to assess these factors and their associations with tick bite preventive practices among Malaysian farmworkers.

**Methods:**

A total of eight cattle, goat and sheep farms in six states in Peninsular Malaysia participated in a cross-sectional survey between August and October 2013

**Results:**

A total of 151 (72.2%) out of 209 farmworkers answered the questionnaire. More than half of the farmworkers (n = 91) reported an experience of tick bites. Farms with monthly acaricide treatment had significantly (P<0.05) a low report of tick bites. Tick bite exposure rates did not differ significantly among field workers and administrative workers. The mean total knowledge score of ticks for the overall farmworkers was 13.6 (SD±3.2) from 20. The mean total tick bite preventive practices score for all farmworkers was 8.3 (SD±3.1) from 15. Fixed effect model showed the effects of four factors on tick bite prevention: (1) farms, (2) job categories (administrative workers vs. field workers), (3) perceived severity of tick bites, and (4) perceived barriers to tick bite prevention.

**Conclusions:**

A high proportion of farmworkers, including administrative workers, reported an experience of tick bites. The effectiveness of monthly acaricide treatment was declared by low reports of tick bites on these farms. Tick bite preventive practices were insufficient, particularly in certain farms and for administrative workers. Our findings emphasise the need to have education programmes for all farmworkers and targeting farms with low prevention practices. Education and health programmes should increase the perception of the risk of tick bites and remove perceived barriers of tick bite prevention.

## Introduction

Ticks are blood sucking arthropods which have been linked to several emerging human and animal diseases worldwide, and they are considered to be of high public health importance [1, 2]. Tick-borne diseases (TBDs) including rickettsioses, anaplasmosis, ehrlichiosis, and borreliosis circulate between wildlife and domestic animals through tick bites. Human beings are the aberrant hosts for ticks [3]. Petney et al [4] documented multiple tick borne pathogens of potential veterinary and human importance in the Southeast Asian ticks. However, information on the disease burden of TBDs and vector-host relationships was still insufficient here. Infectious diseases caused by tick-borne pathogens have been reported in Malaysia [5]. In Malaysia, *Dermacentor*, *Ixodes* and *Haemaphysalis* ticks had been implicated as the reservoirs and potential vectors for rickettsial diseases [6–8]. In a previous investigation, the seroprevalence of spotted fever group rickettsiae amongst febrile patients from rural health centres in Malaysia was 42.5%. This prevalence was significantly higher among those patients who were in the agricultural sectors compared to other occupations [9]. Although the seropositivity rate was not an indication of acute infection, it reflected the extent of exposure of Malaysian rural population to rickettsiae, and indirectly, the exposure of rural populations to tick bites. As ticks harbour more than one pathogen, there is a high possibility for simultaneous transmission of multiple pathogens during tick bites. Antibodies against other tick-borne pathogens such as *Borrelia* and *Coxiella spp*. had been detected from Malaysian patients [10–12]. Neutralising antibodies against Langat virus (a tick borne virus) had been detected in some Malaysian aborigines [13, 14]. A sample with anti-CCHFV immunoglobulin G antibodies was detected from this population recently [15]. Additionally, several cases of tick paralysis have also been reported in Malaysia [16]. Rickettsioses and ehrlichiosis have been reported in Thailand, a neighbouring country of Malaysia. Approximately 20 (44%) out of 50 healthy volunteers in Thailand were seropositive for *E*. *chaffeensis* [17]. Ehrlichial infection is often underdiagnosed as it is often asymptomatic [18, 19]. Worksites with high, wild grass, bushes, and leaf litters are likely to have ticks, and this may pose a high risk for TBDs among outdoor workers, forestry workers, and farmworkers [20–27]. Education for preventive practices is important to reduce the risk of farmworkers from contracting TBDs. Preventive practices for TBDs can be classified into three domains: environment modification, such as landscape management by cutting and removing leaf litters; chemical control measures, for instance, application of acaricides to wild or domestic hosts; and personal protective measures [28]. Nevertheless, landscape and chemical measures are often short-term and may cause severe ecological damage and toxicity for animals and humans. Until further development of more effective tick control practices, personal prevention against tick bites will remain the best measure to prevent TBDs [29]. Studies of ticks and TBDs in many countries indicated that regardless of the awareness of the preventive measures against tick bites, public compliance with tick bite prevention is low [30, 31]. Results of surveys suggest that certain factors, including socio-demographic characteristics, past experience of tick bites, knowledge, and health beliefs may potentially influence personal prevention practices [30, 32–36].

According to the Health Belief Model (HBM), a social cognitive framework for studying health behaviour, the best compliance with preventive measures is consistent by perceiving the severity of a disease, the belief of being at risk, the perceived benefits and self-efficacy of prevention measures, and the perceived low barriers to performing the prevention [25, 35, 37, 38]. Little is known about the prevalence and risk of acquiring TBDs amongst farmworkers in Malaysia; however, various tick borne bacterial and viral pathogens have been described in this country and there is evidence of the exposure to tick bites. Due to emerging (re-emerging) TBDs and to prevent potential outbreaks of tick borne infections in humans or animals, the awareness of people at high risk of potential exposure to tick borne pathogens should be increased. This study was designed to examine the experiences, knowledge, health beliefs, and prevention practices of Malaysian farmworkers towards exposure to ticks, and determine factors that may influence tick bite prevention measures. It is hoped that the findings from this study may provide important insights for the development of necessary educational and preventive programmes for TBDs in this region.

## Methods

### Study population

A cross-sectional study was conducted from August to October 2013 at eight domestic animal farms which were routinely monitored by the Department of Veterinary Services (DVS), Ministry of Agriculture and Agro-Based Industry, Malaysia. Farms were selected through a universal sampling. A total of eight government (DVS) cattle, goat and sheep farms were included in the survey. Farmworkers who were Malaysian citizens and 18 years old and above were invited to participate in the study. Two types of farmworkers were included in this: field workers who have direct contact with animals for at least one day a week, and administrative workers who have little to no contact with farm animals, including secretaries, drivers, and accountants.

### Instrument

The study was conducted using an interviewer-administered questionnaire which included 45 questions covering six aspects. Farmworkers’ socio-demographic characteristics were asked in seven items. Subsequently, farmworkers’ experience of tick bites and getting sick after tick bites were probed (two items). The answer choices which scored “Yes” was given a score of one, and “No” or “Do not know” was scored as zero. In the treatment-seeking section, which was applicable for those who had an experience of tick bite, participants were asked what treatment they had sought (five items). Answer choices were either “Yes” or “No”.

The knowledge about ticks was assessed across several domains: 1) general knowledge about ticks (six items); 2) knowledge about prevention (six items); 3) knowledge about signs and symptoms (six items); and 4) knowledge about treatment (two items). For each question, a correct response was given a score of one, and an incorrect or “Don’t know” was scored as zero. The scores were summed to get a total knowledge score ranging from 0 to 20, with a higher score indicating better knowledge. Participants’ beliefs regarding tick bites and preventive measures were assessed in the third section based on HBM components as a conceptual framework which included perceived severity and susceptibility, perceived benefits, perceived barriers, and perceived self-efficacy (five items) [37]. Responses were based on a 6-point Likert scale (range from 1-“Not at all” to 6-“Extremely”).

Prevention practices were questioned over five items which have been recommended by the U.S. Centers for Disease Control and Prevention (CDC) for outdoor workers. It includes using repellents, checking the body to find ticks, taking a shower, changing and washing the clothes after returning from bushy or high grass areas and dealing with farm animals, and wearing protective clothes (long-sleeved, light-coloured shirts and long pants tucked into socks or boots) [39]. For the analyses, the responses were scored as 0 “Not at all”, 1 “Rarely”, 2 “Sometimes”, and 3 “Often”. The scores were summed to acquire a total prevention practices score ranging from 0 to 15, with higher scores indicating a high performance of preventive practices. Information on the farming management including type of animals, type of grazing and frequency of applying acaricides, and alternative methods to control ticks were obtained from the managers of each farm.

The questionnaire was content validated by a panel of experts comprised of a researcher, physician, epidemiologist, and veterinarian to ensure that the items have acceptable content validity. Each item was rated based on essentiality (essential, useful but not essential, not necessary) and clarity (clear, item needs revision, not clear) on a three-point scale. The content validity ratio (CVR) for each item (which ranges from -1 to 1) was determined. The items with a value greater than zero were retained and the rest were discarded. Then, the mean of CVR value was analysed to get a content validity index (CVI). A CVI greater than 0.6 was considered acceptable [40]. Internal consistency with the overall study sample was conducted. Cronbach’s alpha coefficient was used to measure the internal consistency of knowledge and preventive practices scale scores, with values greater than 0.6 indicating acceptable internal consistency [41].

The questionnaires were translated into Bahasa Malaysia (the national language of Malaysia) as all farmworkers in this study were able to understand Bahasa Malaysia. The translated questionnaires were reviewed by secondary translators and back translation was conducted on the primary translated version. The back-translated version was reviewed by the researchers and necessary edits were made to the target language version. Then, it was pilot tested on 10 farmworkers during the logistics visit of the first farm. The participants stated that the questions were understandable. Overall, participants took approximately 15 minutes to complete the questionnaire. Prior to the survey, an oral briefing on the objective and methodology of the study was given to the participants. Interviewers also showed participants some plastic models of ticks to distinguish them from other arthropods, and they were reminded that their participation in the interview was voluntary. Written informed consent of the participants was obtained and they were assured that their responses would be confidential. Interviewers checked all questions in the questionnaire that have been completed by participants to ensure complete responses to all questions and no missing data.

### Ethic statement

The study was approved by the Medical Ethics Committee, University Malaya Medical Centre, Kuala Lumpur, Malaysia (MEC Ref. No. 968.31).

### Analyses

The data were analysed using SPSS version 20 for Windows software. The comparison of means was analysed via independent samples t-test and one-way analysis of variance (ANOVA). The significance of differences in percentages was analysed by chi-square test.

Tick bite preventive practices score was the outcome variable of interest. Socio-demographic characteristics, an experience of tick bites, knowledge and health beliefs were considered as independent variables. Fixed effects model was used with all government farms were included, to controls for farm level heterogeneity and consider the effect of the farmworkers level predicators [42]. Variables were included in the model if they had a p-value < 0.05 on univariate analysis and a dummy variable was generated for each farm. By including farm dummies as fixed effects, the average differences across farms in any observable or unobservable predictors were controlled. Farms characteristics were excluded from the model since they are constant within each farm. A likelihood-ratio test was used to determine if including more fixed factors are required to improve the analyses.

## Results

### Socio-demographic data of farmworkers

The selected farms were located at the suburban areas of the Southern, Northern, Central and Eastern parts of Peninsular Malaysia. The farms were surrounded by either oil palm or rubber plantation or secondary forests. Management of the farms varied in the type of livestock (cattle, goat and sheep), and grazing systems (rotational or zero). There was difference in the tick management programs in each farm; with most farms having fixed schedule (every 1-, 2-, 6-monthly) for use of acaricide on the farm animals. Three farms reported acaricide treatment of their farm animals only when tick infestation was a high. All the cattle in six farms and the sheep farm were managed by rotational grazing system while the goat farm (farm 5) was kept under zero grazing practice (Table 1). A total of 151 out of 209 farmworkers completed the questionnaire, giving an overall response rate of 72.2%. Table 2 shows the socio-demographic data of the farmworkers. The mean age of the farmworkers was 39.8 years (SD ± 11.1) with a range of 22 to 59 years. The majority were field workers (n = 117, 77.5%). Most of the participants had worked for more than five years in the farm (39.1% between 5 to 15 years; and 35.1% more than 15 years). A large proportion of the farmworkers were Malay (n = 140, 92.7%). About 80% of farmworkers had attained at least a high school education. More than half (n = 85, 56.3%) had less than RM 2000 monthly household income.

**Table 1:** Characteristics of farms (N = 8).

	Farm 1	Farm 2	Farm 3	Farm 4	Farm 5	Farm 6	Farm 7	Farm 8	p value
**Location of farm**	Southern part	Southern part	Northern part	Central part	Southern part	Northern part	East coast	Central part	-
**Type of livestock**									
	Cattle	Cattle	Sheep	Cattle	Goat	Cattle	Cattle	Cattle	-
**Type of grazing system**									
	Rotation	Rotation	Rotation	Rotation	Zero grazing	Rotation	Rotation	Rotation	-
**Frequency of applying acaricides**									
	Monthly	Every 6 months	Only when ticks’ density is high	Every 6 months	Only when ticks’ density is high	Only when ticks’ density is high	Monthly	Every 2 months	-
**No. of farmworkers**									
	N = 41	N = 36	N = 18	N = 23	N = 18	N = 24	N = 28	N = 21	-
**Response rate (%)**									
	71.4	72.2	72.2	73.9	72.2	70.8	71.4	71.4	-
**Farmworkers’ experience of tick bites, n (%)**									
	8(26.7%)	16(61.5)	7(53.8)	13(76.5)	11(84.6)	14(82.4)	12(60.0)	10(66.7)	0.002^‡^**
**Farmworkers’ knowledge mean (SD)**^†^									
	13.1(2.8)	14.3(4.1)	13.2(3.7)	13.4(2.8)	14.1(2.2)	14.2(3.0)	13.5(3.3)	13.1(3.2)	0.84^¶^
**Prevention practice mean (SD)**^††^									
	7.8(3.7)	7.6(3.3)	9.5(3.4)	8.2(2.1)	9.6(3.4)	7.2(1.9)	10.0(2.4)	7.0(2.1)	0.01^¶^*

^†^Total knowledge score (0–20 items score), Mean = 13.6 (SD±3.2)

^††^Total preventive practices score (0–15 items score), Mean = 8.27 (SD±3.09)

^‡^Chi square test.

^¶^ One-way analysis of variance (ANOVA)

Association is significant at the **p < 0.01; *p < 0.05.

**Table 2:** Socio-demographic characteristics of farmworkers, experience of tick bite, total knowledge score & total preventive practices score (N = 151).

	Overall	Experience of tick bite		Total knowledge (0–20 items score)		Total personal preventive practices (0–15 items score)	
	N = 151 N(%)	n = 91 N (%)	p value^‡^	N = 151 Mean (SD)	p value^¶^	N = 151 Mean (SD)	p value^¶^
**Age group (years old)**							
18–35	71(47.0)	37(52.1)		13.6(3.4)		8.4(2.7)	
36–50	44(29.2)	29(65.9)	0.15	13.6(2.7)	0.99	8.5(2.9)	0.43
<51	36(23.8)	25(69.4)		13.7(3.4)		7.7(3.8)	
**Gender**							
Male	121(80.1)	68(56.2)		13.3(3.2)		8.5(2.9)	
Female	30(19.9)	23(76.7)	0.04*	13.2(3.1)	0.43	7.4(3.4)	0.07
**Ethnicity**							
Malay	140(92.7)	84(60)		13.6(3.2)		8.3(3.1)	
Non-Malay^†^	11(7.3)	7(63.6)	0.81	14.0(2.7)	0.68	8.0(3.2)	0.76
**Highest educational level**							
High school & below	121(80.1)	72(59.5)		13.3(3.08)		8.4(3.2)	
Diploma & Tertiary	30(19.9)	19(63.3)	0.70	13.2(3.6)	0.43	7.8(2.7)	0.39
**Years working on farm**							
<5years	39(25.8)	18(46.2)		13.7(3.7)		7.9(2.5)	
5–15	59(39.1)	39(66.1)	0.11	13.4(2.9)	0.83	8.6(3.1)	0.63
>15	53(35.1)	34(64.2)		13.8(3.1)		8.2(3.5)	
**Average monthly household income (MYR)**^††^							
≤RM 2000	85(56.3)	51(60)		13.9(2.8)		8.7(3.0)	
2001–3000	31(20.5)	17(54.8)	0.66	13.3(3.4)	0.28	7.8(3.4)	0.21
> RM3000	35(23.2)	23(65.7)		13.0(3.7)		7.7(2.9)	
**Job categories**							
Administrative workers	34(22.5)	17(50)		13.2(3.9)		6.6(3.1)	
Field workers	117(77.5)	74(63.2)	0.16	13.7(2.9)	0.44	8.7(2.9)	0.001**
**Health beliefs**							
**Perceived severity (scale 1–6)**							
Scale 1–3	117(77.5)	76(65)		13.2(3.1)		7.9(3.1)	
Scale 4–6	34(22.5)	15(44.1)	0.03*	14.9(3.2)	0.007*	9.4(2.8)	0.01*
**Perceived susceptibility (scale 1–6)**							
Scale 1–3	125(82.8)	77(66.6)		13.6(3.1)		8.2(3.2)	
Scale4-6	26(17.2)	14(53.8)	0.46	13.6(3.6)	0.95	8.8(2.1)	0.24
**Perceived benefits (scale 1–6)**							
Scale1-3	45(29.8)	29(64.4)		13.4(3.7)		7.9(3.6)	
Scale4-6	106(70.2)	62(58.5)	0.49	13.7(2.9)	0.55	8.4(2.8)	0.43
**Perceived barriers (scale 1–6)**							
Scale4-6	28(18.5)	15(53.6)	0.42	13.7(3.2)		5.9(2.1)	
Scale1-3	123(81.5)	76(61.8)		13.6(3.2)	0.80	8.8(3.0)	0.001***
**Self-efficacy**							
Scale1-3	68(45.0)	48(70.6)		13.5(3.6)		8.1(3.2)	
Scale4-6	83(55.0)	43(51.8)	0.02*	13.7(2.8)	0.62	8.4(2.9)	0.58
**Knowledge categories**						
0–13	78(51.7)	48(61.5)		-		8.0(3.1)	
14–20	73(48.3)	43(58.9)	0.74	-		8.5(3.1)	0.84
**Experience of tick bite**							
Yes	91(60.3)	-		-		8.4(2.7)	
No/Not sure	60(39.7)	-		-		8.1(3.6)	0.84

^†^ Non-Malay (Chinese, Indian, others)

^††^1 US Dollar = 4.0 Malaysian Ringgit (MYR).

^‡^Chi square test.

^¶^ Independent samples t-test and one-way analysis of variance (ANOVA)

Association is significant at the ***p < 0.001; **p < 0.01; *p < 0.05.

### Experience of tick bites and treatment-seeking behaviour

A total of 91 (60.3%) out of 151 farmworkers reported an experience of tick bites. Table 1 shows that there was significant differences (P < 0.05) in the experience of tick bites among farms, by which the proportion of tick bite experience was high in farm 5 (n = 11, 84.6%) and farm 6 (n = 14, 82.4%), but low in farm 1 (n = 8, 26.7%). Only 37 farmworkers (24.5%) reported feeling sick after a tick bite. There was no significant difference (P = 0.16) in the experience of tick bites among field workers and administrative workers, as 63% (n = 74) of field workers and 50% (n = 17) of administrative workers reported an experience of tick bites. About 32% (n = 29) of farmworkers reported experience of tick bites at their worksites (farm), while 38.5% (n = 35) reported other than their worksites. A total of 17.6% (n = 16) had tick bites after contact with animals; however, about 12% (n = 11) could not recall the places where they had been bitten. About half of the administrative workers (n = 9, 53%) reported that they had been bitten by ticks other than their worksites compared to 35.1% (n = 26) of field workers. The proportion of experience of tick bites at worksites was approximately the same for both administrative workers (n = 5, 29.4%) and field workers (n = 24, 32.4%). A higher proportion of field workers (n = 14, 18.9%) reported tick bites after contact with animals, compared to administrative workers (n = 2, 11.8%). Table 2 shows significant differences (P < 0.05) in the reporting of tick bites according to the gender of the farmworkers. The reporting of tick bites was higher among females (76.7%) than males (56.2%). Tick bites reporting did not differ significantly with other socio-demographic characteristics of the farmworkers. The results in Table 3 demonstrate a statistically significant difference (P<0.05) in the experience of tick bites with the frequency of acaricide treatment on the farm animals. Farmworkers working in a farm with a monthly acaricide treatment had the lowest proportion (n = 20, 40%) of experience of tick bites compared to other farmworkers. A large proportion (n = 32, 74.4%) of farmworkers with experience of tick bites came from farms with irregular schedule of acaricide treatment. There were no significant differences in the proportion experience of tick bites by the type of livestock and grazing system.

**Table 3:** The Experience of tick bites associated with characteristics of farms.

			Experience of tick bite (N = 151)		
	Number of farms	Number of Farm workers	Yes	No/Not sure	
Characteristics of farms	N = 8 N	N = 151 N	n = 91 N (%)	n = 60 N (%)	P value^†^
**Frequency of applying acaricide**					
Monthly	2	50	20(40.0)	30(60.0)	
Every 2months	1	15	10(66.7)	5(33.3)	
Every 6 months	2	43	29(67.4)	14(32.6)	0.004*
Only when the density of ticks are high	3	43	32(74.4)	11(25.6)	
**Type of grazing system**					
Zero grazing	1	13	11(84.6)	2(15.4)	
Rotational grazing	7	138	80(58.0)	58(42.0)	0.06
**Type of livestock**					
Cattle	6	125	73(58.4)	52(41.6)	
Goat	1	13	11(84.6)	2(15.4)	0.16
Sheep	1	13	7(53.8)	6(46.2)	

^†^ Chi square test

*Association is significant at the P<0.05

The treatment seeking preferences of farmworkers who had an experience of tick bites (n = 91, 60.3%) were natural home remedies (n = 50, 54.9%), traditional medicine (n = 45, 49.5%), over-the-counter medications (n = 41, 45.1%) such as pain killers and fever reducers, or visiting a clinic or hospital (n = 36, 39.6%). A total of 25.3% (n = 23) farm workers did not seek any treatment. A higher proportion of field workers (n = 25, 33.8%) reported a visit to a clinic or hospital after the development of signs and symptoms as compared to the administrative workers (n = 3, 17.6%).

### Knowledge

**Fig 1:**
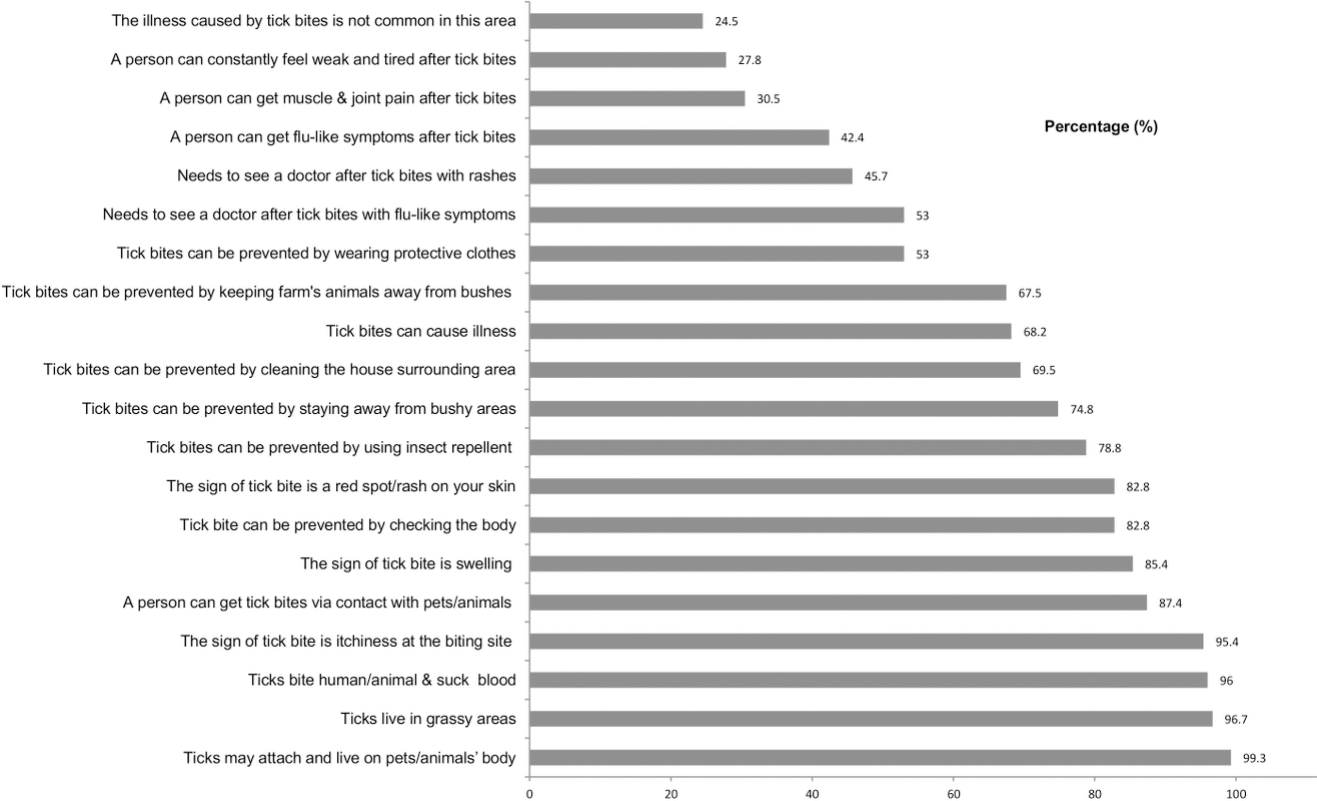
The proportion with correct responses of knowledge related to ticks.

As shown in Fig 1, one third (31.8%) of the farmworkers were unaware of the risk of acquiring diseases from tick bites. The majority knew that the prevention of tick bites could be performed by checking body for ticks after work (82.8%) and wearing protective clothing (53%). Less than half of the farm workers were aware that flu-like symptoms (42.4%), muscle and joint pains (30.5%), and persistent weakness and tiredness (27.8%) were some of the symptoms suggestive of TBDs. The internal consistency of the knowledge items assessed by Cronbach’s alpha was 0.72, thus, showing reasonable internal consistency. The mean total knowledge score of the farmworkers for ticks and tick-borne diseases was 13.6 (SD ± 3.2) out of a possible score of 20. The mean total knowledge score was higher for farmworkers in farm 2 (mean = 14.3, SD ± 4.1), followed by those in farm 6 (mean = 14.2, SD ± 3.0) and farm 5 (mean = 14.1, SD ± 2.2); however, the differences in mean total knowledge score among farms were not significant (Table 1). As shown in Table 2, the mean total knowledge score did not differ significantly with the socio-demographic characteristics of either field workers or administrative workers.

### Health Beliefs

On a 6-point scale where 1 equalled “Not at all” and 6 equalled “Extremely”, the majority of farmworkers (77.5%) stated that tick bites are “Not at all” to “Somewhat not” serious (on a scale of 1 to 3, mean = 2.7; SD ± 1.2). A significantly higher proportion of those who had at least a high school education attainment (n = 99; 81.8%) perceived the severity of tick bites on a scale of 1 to 3 (P < 0.05). Perceived severity of tick bites did not differ significantly with other socio-demographic characteristics of the farmworkers.

As shown in Table 2, statistically significant differences (P < 0.05) were observed in the perceived severity of tick bites by experience of tick bites and mean total knowledge score of ticks. Farmworkers who indicated that tick bites are “Not at all” to “Somewhat not” serious (scale 1 to 3) had a significantly more experience of tick bites (n = 76; 65%) and a lower level of mean total knowledge score of ticks (mean = 13.2; SD ± 3.1) versus those who said tick bites are “Somewhat” to “Extremely” serious on a scale of 4 to 6.

A large proportion of farmworkers (82.8%) also cited “Extremely low” to “Somewhat low” susceptibility to getting tick bites (scale of 1 to 3, mean = 2.6; SD ± 1.1). Regarding perceived benefits and barriers to tick bite prevention, the majority (70.2%) stated that prevention practices are “Somewhat beneficial” to “Extremely beneficial” (scale of 4 to 6, mean = 4.1; SD ± 1.4), and a high proportion (81.5%) also mentioned there were “Not at all” to “Somewhat no” barriers to practicing tick bite prevention (on a scale of 1 to 3, mean = 2.7; SD ± 1.1). The perceived susceptibility to getting tick bites, and the perceived benefits and barriers to tick bite prevention did not differ significantly with socio-demographic characteristics, experience of tick bites, or mean total knowledge score of ticks (Table 2).

About half (55%) of the farmworkers perceived “Somewhat” to “Extremely” self-efficacy in preventing tick bites (scale of 4 to 6, mean = 3.7; SD ± 1.3). The perceived self-efficacy of tick bites prevention did not differ significantly with socio-demographic characteristics and mean total knowledge score of ticks. As shown in Table 2, a statistically significant difference (P < 0.05) was observed in the perceived self-efficacy of tick bite prevention by experience of tick bites. Farmworkers who believed “Not at all” to “Somewhat not” self-efficacy in preventing tick bites (scale of 1 to 3), reported a higher proportion of experience of tick bites (70.6%) than those who said “Somewhat” to “Extremely” self-efficacy of tick bite prevention (51.8%). The health beliefs did not differ significantly among field workers and administrative workers.

### Prevention practices

**Fig 2:**
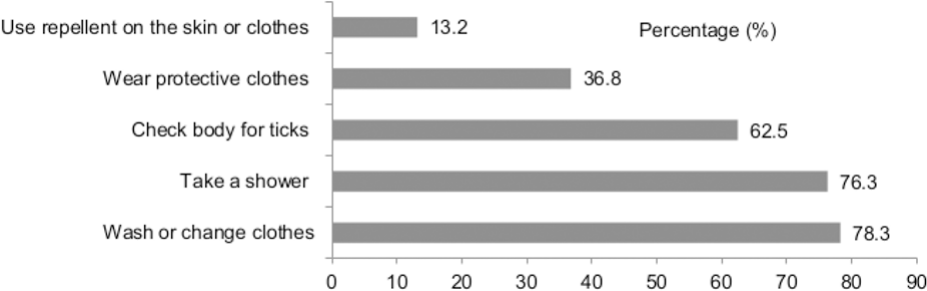
The proportion of personal preventive practices (answer choices Sometimes/Often).

Regarding the protection against tick bites in bushy, high grass areas or worksites (farm), the majority of the farmworkers stated that they “Sometimes/Often” wash or change clothes (86.1%), take a shower (84.8%), and check their body for ticks (62.9%). However, only one third (36.4%) of the farmworkers indicated that they wore protective clothes including long-sleeved, light-coloured shirts with long pants tucked into socks or boots. A minority (11.3%) stated that they used repellent on the skin or clothes (Fig 2). Cronbach’s alpha coefficients for personal prevention practice items was 0.69, indicating adequate internal consistency.

The mean total tick bite preventive practices score for all farmworkers was 8.27 (SD ± 3.09) out of a possible score of 15. There were statistically significant differences (P < 0.05) in mean total tick bite preventive practices score among farms. The score was higher in farm 7 (mean = 10.0, SD ± 2.4), followed by farm 5 (mean = 9.6, SD ± 3.4) and farm 3 (mean = 9.5, SD ± 3.4) and the lowest in farm 8 (mean = 7.0, SD ± 2.1) (Table 1). As shown in Table 2, field workers had a significantly higher (P < 0.05) mean total tick bite preventive practices score (mean = 8.7; SD ± 2.9) compared to administrative workers (mean = 6.6; SD ± 3.1). Likewise, the mean total tick bite preventive practices score was significantly higher (P < 0.05) among farmworkers who indicated that tick bites are “Somewhat” to “Extremely” serious on a scale of 4 to 6 (mean = 9.4; SD ± 2.8) and there are “Not at all” to “Somewhat no” barriers to practicing tick bite prevention on a scale of 1 to 3 (mean = 8.8; SD ± 3.0), versus the perceived severity on a scale of 1 to 3 (mean = 7.9; SD ± 3.1), and perceived barriers on a scale of 4 to 6 (mean = 5.9; SD ± 2.1), respectively.

Fixed effects model analysis of factors associated with total tick bite preventive practices was conducted. The model was consisted of fixed effects (farm dummies and significant predictors in univariate analysis) and controlled for the average differences across farms that might affect the predictors within each farm. The model revealed the effects of farms, job categories, perceived severity and perceived barriers on tick bite preventive practices. The results in Table 4 show that farmworkers in farm 5 (β = 2.31, P < 0.05) and farm 7 (β = 3.31, P < 0.001) had higher scores of tick bite preventive practices compared to the reference group (farm 8). Administrative workers had lower (β = -2.16, P < 0.001) tick bite preventive practice score compared with the field workers. Farmworkers who perceived tick bites “Not at all” to “Somewhat not” serious (scale of 1 to 3) had lower scores of tick bite preventive practices (β = -1.37, P < 0.05) compared with the reference group (scales of 4 to 6). Likewise, farmworkers who were of the opinion that there were “Somewhat” to “Extremely” barriers to tick bite prevention (scale of 4 to 6) had a low score (β = -2.61, P < 0.001) for tick bite prevention when compared to the reference group (scales of 1 to 3).

**Table 4:** Fixed effect estimates of factors associated with total tick bite preventive practices score.

Variables	β	SE^†^	T test	95%CI^††^
**Job categories**				
Administrative workers	-2.16***	0.53	-4.08	-3.21–1.11
Field workers	Reference			
**Perceived severity (scale 1–6)**				
Scale 1–3	-1.368*	0.53	-2.58	-2.42—.32
Scale 4–6	Reference			
**Perceived barriers (scale 1–6)**				
Scale4-6	-2.61***	.58	-4.53	-3.75–1.47
Scale1-3	Reference			
**Farms of survey**				
Farm1	0.95	0.83	1.15	-0.684–2.586
Farm2	0.92	0.85	1.08	-0.762–2.593
Farm3	1.84	0.99	1.85	-0.125–3.798
Farm4	1.33	0.95	1.40	-0.542–3.197
Farm5	2.31*	0.99	2.32	0.342–4.270
Farm6	0.95	0.93	1.02	-0.893–2.787
Farm7	3.31***	0.89	3.71	1.547–5.069
Farm8	Reference	Reference	Reference	Reference

^†^Standard Error

^††^95% confidence interval

Association is significant at the ***p < 0.001; *p < 0.05.

R^2^ = 0.34, adjusted R^2^ = 0.29

According to likelihood-ratio test, the model that included variables with P < 0.05 in univariate analysis (farm dummies, job categories, perceived severity and perceived barrier) significantly improved the prediction compared to the model with only farm dummies. A likelihood-ratio chi-square indicated that the change in -2 restricted log likelihood of 43.68 (df = 2) was statistically significant, P < 0.01. The adjusted R^2^ value of the model with only farm dummies was 0.07, indicating that 7% of the variance in the model was explained by the variability between the farms. The increase in the adjusted R^2^ value (from 7% in model with only farm dummies to 29% in model with job categories, perceived severity, perceived barrier and farm dummies) indicates that the later model explains more variance.

## Discussion

To the best of our knowledge, there is a lack of data on the prevalence of tick bites and TBDs among farmworkers in Southeast Asian region including Malaysia. About 60% of farmworkers reported an experience of tick bites in this study. Tick exposure of farmworkers has been reported in other parts of the world. Findings of a study in north-west Turkey showed that about 52% of farmworkers had an experience of tick bites [43]. A high proportion (87%) of farmers in Eastern Poland who had contact with animals also reported frequent tick bites [22].

The results in Table 1 indicate that experience of tick bites was significantly different among farms, which farm 1 had the lowest proportion (26.7%) of reporting tick bites and farm 5 had the highest proportion (84.6%). A set of reasons can be attributed to the disparity among farms in the reports of tick bites. Findings showed that the frequency of acaricide treatment was correlated to the number of reports of tick bites (Table 3). Experience of tick bites was the lowest in farms with monthly acaricide treatment such as farm 1 and the highest in farms with irregular schedule for acaricide treatment such as farm 5. A survey of TBDs in cattle in western Kenya reported that irregular acaricide application treatment of farm animals could be a factor for the higher prevalence and incidence of TBDs in cattle [44]. Accordingly, irregular use of acaricides can increase the exposure to ticks for farmworkers. It was suggested that farm authorities should attempt a regular monthly acaricide application to reduce tick abundance on farms.

Adopting the zero grazing system is another method to control farm animals from being exposed to ticks, and increasing freedom of grazing will increase incidences of TBDs [45, 46]. However, as shown in Table 1, a high proportion (84.6%) of farmworkers from farm 5 (the sole farm which is implementing zero grazing system) reported an experience of tick bites as compared to other farms with rotational grazing. It is possible that farmworkers might have exposed to ticks from bushy or high grass areas while cutting and carrying fodder to farms. The result recommends increasing awareness among farmworkers regarding the risk of tick bites exposure in bushy or high grass areas, in addition to contact with animals.

The findings illustrate that regardless of contact with farm animals, both field workers and administrative workers were exposed to tick bites. Most of the surveyed farms in this study were surrounded with high grasses and vegetation which were the natural habitat for some ticks. These findings imply that all farmworkers regardless of their job categories should be informed of the risk of tick bites. Except for the female farmworkers who reported experiences of tick bites more frequently than the male farmworkers, there is no correlation in the reported experience of tick bites with the age, ethnicity, educational level, years of working on the farm, and average monthly household income of the farmworkers (Table 2).

The mean total knowledge score (13.6 ± 3.2 out of a possible score of 20) of the farmworkers on ticks and TBDs reflects a moderate knowledge concerning ticks and TBDs. The mean total knowledge score of field workers was slightly higher than the administrative workers, suggesting that administrative workers should be also educated about ticks and TBDs, as they had the similar risk of getting tick bites. One third of the farmworkers were not aware that tick bites could cause diseases, and many stated that TBDs were not common in their areas. More than half of the farmworkers were not able to relate the common symptoms of TBDs. As tick bites symptoms were unknown to many, this could perhaps result in having relatively few farmworkers reporting feeling sick after tick bites. This finding implies that farmworkers need awareness intervention that tick bites can cause infections. Further, it was found that treatment seeking preferences of the farmworkers for tick bites were mainly home remedies and traditional medicine. Some were unaware of the availability of treatment for tick bites and few visited clinic and hospital for treatment. In a qualitative study regarding dengue fever in Malaysia participants reported the use of traditional medicine and home remedies beside seeking modern medical treatment for suspected dengue, as they were of the opinion that the ‘‘natural” origins of home remedies and traditional medicine were helpful and harmless [47]. Therefore, comprehensive educational and health programmes for tick bite prevention among farmworkers should include imparting knowledge about tick bites, signs and symptoms and health implications of TBDs, as well as recommendation to seek medical attention if they develop symptoms after getting tick bites.

With regards to health beliefs in the context of HBM, the majority of the farmworkers had a low perceived severity of tick bites. The results showed that lower perceived severity of tick bites was significantly correlated to lower level of educational attainment and lower knowledge score of ticks. Other studies implicated that a lack of knowledge on ticks and TBDs can make people underestimate the risk of tick bites [38, 48]. Therefore, to augment the perceived severity of tick bites, an education intervention to improve knowledge of ticks and TBDs is required, particularly for low educated farmworkers. Moreover, low perceived severity was significantly associated with more experience of tick bites, as demonstrated in this study (Table 2). On the one hand, farmworkers could underestimate the risk due to a low perceived severity of tick bites which resulted in more tick exposure and tick bites. On the other hand, farmworkers perceived tick bites are not a serious health problem as they did not experience major health problems after tick bites. This could be due to the lack of information on the health impacts of TBDs. Farmworkers should be taught that tick bites warrant careful attention, since various tick borne bacterial and viral pathogens have been described in the local setting [49]. The low perceived self-efficacy of prevention was significantly correlated with experience of tick bites. This implies that the low self-efficacy of prevention might lead to less engagement in the prevention measures, and subsequently more experience of tick bites. A mixed method study of the public and experts about predictors of preventive behaviour against tick bites in the United Kingdom showed that self-efficacy of tick checking preventive behaviour played a strong role in determining whether that behaviour would be adopted [50]. Therefore, an effective education should place more emphasis on increasing self-efficacy of preventive behaviour to encourage compliance with tick bite prevention actions.

The tick bite preventive measures taken by farmworkers in this study were considered as moderate based on a mean total tick bite preventive practices score of 8.3 (SD ± 3.1) out of a possible score of 15. The most common prevention measures taken by farmworkers were washing or changing clothes (86.1%), and taking a shower after coming back from woody, bushy, or high grass areas or dealing with farm animals (84.8%). In contrast, wearing protective clothing (36.4%) and using repellent on the skin or clothes (11.3%) were less performed prevention measures. A survey of public perception regarding Lyme disease recommended that less compliance with wearing protective clothes could be due to the climate and belief that wearing protective clothing is overdoing prevention [38]. Due to hot and humid weather throughout the year in Malaysia, it is expected that farmworkers are less willing to wear protective clothing such as that suggested by CDC with long-sleeved, light-coloured shirts and long pants tucked into socks or boots [39]. A study of outdoor workers from North Carolina’s Divisions of Parks and Recreation, Forestry, and Wildlife showed that long-lasting permethrin impregnated uniforms are highly effective for at least one year in deterring tick bites in the context of typical tick bite prevention measures employed by outdoor workers [51]. Although most of the farmworkers were aware of the possibility of using repellent on skin or clothes, repellents were not widely used. This could be due to the high cost of repellents and that repellents are not available for the workers. Thus, it is important to encourage farmworkers through education to improve the uptake of protective clothing and applying repellent during work on the farm or in bushy areas. Farms’ authorities can provide an appropriate uniform for farmworkers like long-sleeved, light-coloured shirts, long pants and boots, as well as offer repellent for farmworkers.

The fixed effect model revealed the effects of farms, job categories and two constructs of HBM (perceived severity of tick bites and perceived barriers to performing prevention), on tick bite preventive practices. There was significant variation among farms regarding tick bite prevention. Farmworkers in farm 5 and 7 had a high tick bite prevention score compared to farm 8. Regardless of practicing tick bite prevention by farmworkers, high proportion of tick bites experiences for instance in farm 5 (Table 1), warrant an assessment of effectiveness of performed prevention measures. The farms’ authorities should address the shortcomings of performance of tick bite prevention and attempt to overcome them. Farmworkers of farms with low tick bite prevention practices should be educated to engage in tick bite prevention practices. The results also showed that field workers were more likely to take actions against tick bites, although all farmworkers, whether field workers or administrative workers, were at risk of getting tick bites. Thus, any educational programme should convey the awareness that all farmworkers are at risk of being bitten by ticks and should adopt personal prevention practices. Farmworkers who perceived a low severity of tick bites and high barriers to tick bite prevention were less motivated to practice tick bite preventive measures. This implies that educational intervention should target a change of farmworkers’ health beliefs and encourage them to engage in tick bite preventive behaviours. Farmworkers must understand that the risks of tick bites and TBDs exceed the difficulties generated by performing prevention actions against ticks. Farms’ authorities should try to distinguish and eliminate barriers which discourage workers to take prevention measures like uncomfortable protective clothing.

The findings of this study should be interpreted with caution. Firstly, the cross-sectional design of the study means a cause and effect relationship of the results is difficult to establish. Secondly, the self-reporting nature of the data may be subjected to reporting bias towards socially desirable responses and behaviours. Thirdly, this survey reflects responses from government farmworkers in Peninsular Malaysia, which may limit the generalisability of results to overall farmworkers in Malaysia. Serological evaluation of TBDs among farmworkers to confirm the self-reporting of tick bites is necessary. Variation across farms regarding tick bites experiences recommends further investigation. These findings provide useful information that we believe would be valuable in guiding government officials in the development of programmes and activities to initiate prevention against tick bites.

## Conclusion

The findings of this study shows that a high proportion of farmworkers, including administrative workers, have experience of tick bites. The reports of tick bites were higher in farms with irregular acaricide treatment of farm animals compared to farms with monthly acaricide treatment, which confirms the effectiveness of monthly acaricide treatment. Tick bite preventive practices were insufficient, particularly for administrative workers. Factors such as farms, perceived severity of tick bites, perceived barriers to perform tick bite prevention, and farmworkers’ job categories were the main determinants of tick bite preventive practices. Therefore, education and health promotion activities should aim farms with low tick bite prevention practices. Education programs should be designed to specifically enhance the perception of the risk of tick bites and address the barriers to prevention to improve the uptake of prevention practices against tick bites. All workers on the farm, including field workers and administrative workers, should be the target of education.

## Supporting Information

S1 STROBE Checklist Checklist for cross-sectional studies. (DOCX)

S1 Dataset Dataset for relevant study. (SAV)
