# Supplementary material for: Factors Associated with Tick Bite Preventive Practices among Farmworkers in Malaysia
Source: PLoS One. 2016 Jun 24;11(6):e0157987. doi: 10.1371/journal.pone.0157987 (PMC4920353; doi:10.1371/journal.pone.0157987)
Supplement: S1 STROBE Checklist — (DOCX) [file pone.0157987.s001.docx]

S1 STROBE checklist - Checklist of items that should be included in reports of ***cross-sectional studies***

|  | Item No | Recommendation |
| --- | --- | --- |
| **Title and abstract** | 1 | (*a*) Indicate the study’s design with a commonly used term in the title or the abstract  Line 29: cross-sectional survey |
|  |  | (*b*) Provide in the abstract an informative and balanced summary of what was done and what was found  Indicated in abstract |
| Introduction | | |
| Background/rationale | 2 | Explain the scientific background and rationale for the investigation being reported  Indicated in introduction |
| Objectives | 3 | State specific objectives, including any prespecified hypotheses  Line 95. This study was designed to examine the experiences, knowledge, health beliefs, and prevention practices of Malaysian farmworkers towards exposure to ticks, and then assess the factors that may influence the tick bite prevention measures. |
| Methods | | |
| Study design | 4 | Present key elements of study design early in the paper  Line 102. A cross-sectional study was conducted |
| Setting | 5 | Describe the setting, locations, and relevant dates, including periods of recruitment, exposure, follow-up, and data collection  Line102: August to October 2013  Line 102-106: eight domestic animal farms which were routinely monitored by the Department of Veterinary Services (DVS), Ministry of Agriculture and Agro-Based Industry, Malaysia. Farms were selected through a universal sampling. A total of eight government (DVS) cattle, goat and sheep farms were included in the survey.  107. field workers who have direct contact with animals  108. administrative workers who have little to no contact with farm animals  111. Instrument |
| Participants | 6 | (*a*) Give the eligibility criteria, and the sources and methods of selection of participants  Line 104: Farms were selected through a universal sampling. All government (DVS) cattle goat and sheep farms were included in the survey.  106: Farmworkers who were Malaysian citizens and 18 years old and above were invited to participate in the study |
| Variables | 7 | Clearly define all outcomes, exposures, predictors, potential confounders, and effect modifiers. Give diagnostic criteria, if applicable  Line 173-181 |
| Data sources/ measurement | 8* | For each variable of interest, give sources of data and details of methods of assessment (measurement). Describe comparability of assessment methods if there is more than one group  Indicated in methodology |
| Bias | 9 | Describe any efforts to address potential sources of bias  105: All government (DVS) cattle, goat and sheep farms were included in the survey.  The study was conducted using an interviewer-administered questionnaire.  Line 167: Prior to the survey, an oral briefing on the objective and methodology of the study was given to the participants. Interviewers also showed participants some plastic models of ticks to distinguish them from other arthropods.  175: Fixed effects model was used with all government farms were included, to controls for farm level heterogeneity and consider the effect of the farmworkers level predicators |
| Study size | 10 | Explain how the study size was arrived at  Line 102: eight domestic animal farms which were routinely monitored by the Department of Veterinary Services (DVS), Ministry of Agriculture and Agro-Based Industry, Malaysia. Farms were selected through a universal sampling.  line 106: Farmworkers who were Malaysian citizens and 18 years old and above were invited to participate in the study. |
| Quantitative variables | 11 | Explain how quantitative variables were handled in the analyses. If applicable, describe which groupings were chosen and why  Indicated in analyses.  Line 170: The comparison of means was analysed via independent samples t-test and one-way analysis of variance (ANOVA). The significance of differences in percentages was analysed by chi-square test.  175: Fixed effects model was used with all government farms were included, to controls for farm level heterogeneity and consider the effect of the farmworkers level predicators |
| Statistical methods | 12 | (*a*) Describe all statistical methods, including those used to control for confounding  Fixed effect model analysis addressed confounding. R^2^=0.34, adjusted R^2^=0.29 have been reported below the table 4.  Line 175-181 |
|  |  | (*b*) Describe any methods used to examine subgroups and interactions |
|  |  | (*c*) Explain how missing data were addressed  Line 162: Interviewers checked all questions in the questionnaire that have been completed by participants to ensure complete responses to all questions and no missing data. |
|  |  | (*d*) If applicable, describe analytical methods taking account of sampling strategy |
|  |  | (*e*) Describe any sensitivity analyses  Not applicable |
| Results | | |
| Participants | 13* | 1. Report numbers of individuals at each stage of study—eg numbers potentially eligible, examined for eligibility, confirmed eligible, included in the study, completing follow-up, and analysed   Line 202: A total of 151 out of 209 farmworkers completed the questionnaire, giving a response rate of 72.2%. |
|  |  | (b) Give reasons for non-participation at each stage  Not eligible to participate |
|  |  | 1. Consider use of a flow diagram   none |
| Descriptive data | 14* | (a) Give characteristics of study participants (eg demographic, clinical, social) and information on exposures and potential confounders  Provided in table 1 and 2 and indicated in results |
|  |  | (b) Indicate number of participants with missing data for each variable of interest  None |
| Outcome data | 15* | Report numbers of outcome events or summary measures  Indication in the tables and results |
| Main results | 16 | (*a*) Give unadjusted estimates and, if applicable, confounder-adjusted estimates and their precision (eg, 95% confidence interval). Make clear which confounders were adjusted for and why they were included\  Indicated 95% CI in table 4. Confounders were taken into account in the fixed effect model |
|  |  | 1. Report category boundaries when continuous variables were categorized   Indicated in results |
|  |  | (*c*) If relevant, consider translating estimates of relative risk into absolute risk for a meaningful time period |
| Other analyses | 17 | Report other analyses done—eg analyses of subgroups and interactions, and sensitivity analyses  Not applicable |
| Discussion | | |
| Key results | 18 | Summarise key results with reference to study objectives  421. The fixed effect model revealed the effects of farms, job categories and two constructs of HBM (perceived severity of tick bites and perceived barriers to performing prevention), on tick bite preventive practices. |
| Limitations | 19 | Discuss limitations of the study, taking into account sources of potential bias or imprecision. Discuss both direction and magnitude of any potential bias  Line 441-448: Firstly, the cross-sectional design of the study means a cause and effect relationship of the results is difficult to establish. Secondly, the self-reporting nature of the data may be subjected to reporting bias towards socially desirable responses and behaviours. |
| Interpretation | 20 | Give a cautious overall interpretation of results considering objectives, limitations, multiplicity of analyses, results from similar studies, and other relevant evidence  Indicated: The findings of this study should be interpreted with caution. |
| Generalisability | 21 | Discuss the generalisability (external validity) of the study results  446. This survey reflects responses from government farmworkers in Peninsular Malaysia, which may limit the generalisability of results to overall farmworkers in Malaysia. |
| Other information | | |
| Funding | 22 | Give the source of funding and the role of the funders for the present study and, if applicable, for the original study on which the present article is based  Indicated; The study is funded by University of Malaya Research Grant (UMRG, RP013-2012 A and E), and Postgraduate Research Grant, University of Malaya (PG026-2012B). Funder role is just providing financial means to conduct the study. |

*Give information separately for exposed and unexposed groups.

**Note:** An Explanation and Elaboration article discusses each checklist item and gives methodological background and published examples of transparent reporting. The STROBE checklist is best used in conjunction with this article (freely available on the Web sites of PLoS Medicine at http://www.plosmedicine.org/, Annals of Internal Medicine at http://www.annals.org/, and Epidemiology at http://www.epidem.com/). Information on the STROBE Initiative is available at www.strobe-statement.org.
